# Domestic Coercive Control and Common Mental Disorders among Women in Informal Settlements in Mumbai, India: a Cross-sectional Survey

**DOI:** 10.1177/08862605211030293

**Published:** 2021-07-30

**Authors:** Suman Kanougiya, Nayreen Daruwalla, Lu Gram, Muthusamy Sivakami, David Osrin

**Affiliations:** 1 Tata Institute of Social Sciences (TISS), Mumbai, Maharashtra, India; 2 SNEHA (Society for Nutrition, Education and Health Action), Mumbai, Maharashtra, India; 3 Institute for Global Health, University College London, UK

**Keywords:** Coercive control, domestic violence, common mental disorder, India

## Abstract

Coercive control behaviors central to the abuse of power appear more frequent than other types of domestic violence, but little is known about its frequency, features, and consequences for women in India. We aimed to examine the prevalence of domestic coercive control and its association with physical, sexual, and emotional domestic violence in the preceding year and symptoms of depression, anxiety, and suicidal thinking. In a crosssectional survey, we interviewed 4,906 ever-married women aged 18-49 years living in urban informal settlements in Mumbai, India. We developed a 24-item scale of coercive control, assessed physical, sexual, and emotional violence using existing questions, and screened for symptoms of depression with the Patient Health Questionnaire (PHQ9), anxiety with the Generalized Anxiety Disorder (GAD7) questionnaire, and suicidal thinking with questions developed by the World Health Organization. Estimates involved univariable and multivariable logistic regression models and the prediction of marginal effects. The prevalence of domestic coercive control was 71%. In total, 23% of women reported domestic violence in the past 12 months (emotional 19%, physical 13%, sexual 4%). Adjusted models suggested that women exposed to controlling behavior had greater odds of surviving emotional (aOR 2.1; 95% CI 1.7, 2.7), physical (1.4; 1.0, 1.9), and sexual (1.8; 1.1, 3.0) domestic violence in the past 12 months; and higher odds of a positive screen for moderate or severe depression (1.7; 1.3, 2.2), anxiety (2.1; 1.3, 3.1), and suicidal thinking (1.7; 1.2, 2.3), and increased with each additional indicator of coercive control behavior. When women reported 24 indicators of coercive control, the adjusted predicted proportion with moderate or severe depressive symptoms was 60%, anxiety 42%, and suicidal thinking 17%. Inclusion of coercive control in programs to support domestic violence, would broaden our understanding of domestic abuse to resemble most victims experience and improve interventions.

## Background

Preventing domestic violence against women is a global imperative (UN Assembly, 2000). Beginning to address it involves understanding that it is a constellation of behaviours, central to which is the abuse of power within the home. Most research has focused on physical and sexual violence and emotional and economic violence to a lesser degree (Howard et al., 2013; Kalokhe et al., 2016). However, bound up with these forms of violence is controlling behaviour in which family members use threats and violence to assert power over the survivor, who suffers negative consequences for non-compliance.

Coercive control involves abusers using a range of means to “hurt, humiliate, intimidate, exploit, isolate, and dominate their victims”(Stark, 2007). These include restricting or controlling movement and access to family, friends, neighbours, and broader social circles. Perpetrators often use gender norms to constrain women’s mobility, time, spending, socialising, and diet. Women may, for example, be compelled to do household chores in a particular way or keep records of expenditure, the processes becoming normalised within gendered expectations to a point at which it is difficult to differentiate the coercive from the normative (Bishop & Bettinson, 2017). The environment reflects, entrenches, and exaggerates social and gender norms and women’s subordinate position in society (Williamson, 2010), potentially to the extent that it is not perceived as abusive. Coercive control tactics are often interpreted as expressions of care, affection, and love, rather than jealousy or proprietariness (Beck & Raghavan, 2010; Dutton et al., 2006; Stark & Hester, 2019; Suarez, 1994; Wolford-Clevenger et al., 2017).

Although survivors may not consider coercive controlling behaviour to be abuse (Richardson et al., 2020), it is more frequent than other types of domestic violence (Kelly & Johnson, 2008). Across countries, between 21% and 90% of women have experienced controlling behaviour from a partner (Antai, 2011; García-Moreno et al., 2005; Graham-Kevan & Archer, 2003; Johnson, 2006; Krantz & Vung, 2009; Mandal & Hindin, 2013; Sapkota et al., 2016). Such behaviour is not limited to specific conflicts or situations. It often manifests early in a relationship and escalates over time (Crossman et al., 2015; Hardesty et al., 2015). Most—but not all—cases include other forms of abuse such as physical or sexual violence (Anderson, 2008; Lischick, 1999). Studies suggest that coercive control can precede, motivate, or increase the likelihood of other types of violence in relationships (Aizpurua et al., 2021; Antai, 2011; Dalal & Lindqvist, 2010; Graham-Kevan & Archer, 2003; Hardesty et al., 2015; Howard et al., 2013; O’Leary et al., 1994; Stark, 2012), particularly when controlling behaviour does not achieve the desired effect (Tanha et al., 2009). There is evidence from studies in India and elsewhere that partners who use coercive control are between three and eight times more likely to perpetrate physical or sexual violence than partners who use physical violence alone (Kelly & Johnson, 2008; Mukherjee & Joshi, 2019; Ram et al., 2019), and that cases involving coercive control are more likely to result in serious harm than cases that involve discrete acts of physical violence (Campbell et al., 2003; Dobash & Dobash, 2015; Myhill & Hohl, 2016; Stark, 2007).

Coercive control appears to be common in India, affecting approximately 50% of women of reproductive age (International Institute for Population Sciences & ICF, 2017; Mukherjee & Joshi, 2019; Richardson et al., 2020). The prevalence estimated from the fourth National Family Health Survey (NFHS-4) was higher in rural than in urban areas and among women with less education and poorer socioeconomic position. Women who reported more instances of controlling behaviour reported higher rates of emotional (54% compared with 5%), physical (64% compared with 17%), and sexual violence (30% compared with 2%) than women who did not(International Institute for Population Sciences (IIPS) & ICF, 2017).

Coercive control harms mental health (Follingstad et al., 1990; Tolman, 1992). A range of symptoms has been described(Dutton et al., 1997; Follingstad et al., 1991), including distress (Richardson et al., 2020) and common mental disorders such as anxiety and depression (Abbott et al., 1995; Bergman & Brismar, 1991; Dutton et al., 1999; Leone, 2010; Richardson et al., 2020; Williamson, 2010; Wolford-Clevenger et al., 2017). Although some studies have examined coercive control in India (Dalal & Lindqvist, 2010; Kalokhe et al., 2018; Mukherjee & Joshi, 2019; Pandey et al., 2009; Ram et al., 2019), knowledge of the implications for mental health is limited (Richardson et al., 2020; Varma et al., 2007). We wanted to understand women’s experiences in urban informal settlements and the risk of harm to their mental health. Our objectives were to examine (1) the prevalence of coercive control, (2) its associations with other forms of domestic violence, and (3) its relationship with depression, anxiety, and suicidal thinking. We hypothesized that coercive control would be common, that it would be associated with other forms of abuse, and that it would be a risk factor for depression, anxiety, and suicidal thinking.

## Methods

### Setting

The study was conducted in urban informal settlements in Mumbai. Informal settlements (slums) are characterised by overcrowding and unsanitary, unhealthy, and dehumanising living conditions. They are subject to insecure land tenure, lack of access to safe drinking water, sanitation, drainage, solid waste management, internal and approach roads, street lighting, education and health care, and low-quality shelter (Chandramouli, 2011; United Nations Human Settlements Programme (UN-Habitat), 2003). A significant proportion of slum dwellers face social burdens and health problems worse than their non-slum and rural counterparts.

### Design

We used data from a survey done before a community-based intervention to prevent violence against women implemented by the non-government organisation SNEHA (Society for Nutrition, Education and Health Action), which has run a program focusing on primary, secondary, and tertiary prevention of violence for 20 years in informal settlements in Mumbai (Daruwalla et al., 2019). The cross-sectional systematic random sample survey included 50 clusters of equal size (~100 respondents from ~500 residential households) in two sizeable informal settlement areas.

### Participants

We interviewed 5122 women aged 18-49 years in a survey designed to understand domestic violence perpetrated by intimate partners and other family members. For this analysis, we limited the dataset to 4906 ever-married women.

### Data Collection

We followed WHO guidelines for research on domestic violence against women (WHO, 2012) and on sexual violence (Jewkes et al., 2012). Details of data collection are available elsewhere (Daruwalla et al., 2020). Briefly, 16 women interviewers with graduate education and three months of training mapped the study areas and visited households to enumerate residents and list potential respondents. From a random starting point in each cluster, alternate households were selected without replacement until we had collected information from 100 women aged 18-49 years (Daruwalla et al., 2020). Because younger women with a disability may be at higher risk of domestic violence (Ellsberg & Heise, 2005), when more than one potential respondent was available in a household, an algorithm led the investigators to select the youngest disabled, youngest married, or youngest unmarried woman. Interviews were arranged in advance to maintain privacy, with a provision for up to three repeat visits. Participants were given a participant information sheet, discussed the nature of the interview and right to withdraw, and gave signed consent. The interview protocols included safety assessment, counselling, liaison with healthcare, police, and legal services, and developing follow-up plans for the survivor and her family (all of these with permission from the survivor). Interviewers used electronic tablets to enter information in a database in CommCare (www.dimagi.com).

### Variables

#### Sociodemographic variables

Marital status was described by a categorical variable distinguishing married respondents from respondents who had been widowed, separated, or divorced. Socioeconomic position was described by quintiles of a standardised score derived from the first component of a principal components analysis of the ownership of 22 assets (Filmer & Pritchett, 2001; Vyas & Kumaranayake, 2006).

#### Exposure: Coercive Controlling Behaviour

Measures of coercive control have not yet been validated in India or elsewhere (Beck & Raghavan, 2010; Dutton et al., 2006; Kirkwood & Gilbert, 1995; Tanha et al., 2009), and questionnaires about psychological abuse do not clearly differentiate coercive control from psychological abuse (Dekeseredy & Schwartz, 2011; Dutton et al., 2006; Myhill, 2015; Straus et al., 1996). Therefore, we developed a 24-item domestic coercive control questionnaire based on programme experience supporting violence survivors, augmented by four focus group discussions with counsellors, community actors, and lawyers. We harmonized items as far as possible with existing questions available in Demographic and Health Surveys and other studies. The categories of response to each question were *no*, *sometimes*, or *all the time*. Taking a conservative approach, we coded response as indicating the experience of an item of coercive control if a woman said that she had suffered it *all the time*. In terms of numbers of different tactics rather than frequency, we described domestic coercive control intensity in a summative variable with values from 0 to 24.

#### Outcomes

Participants were screened for symptoms suggestive of depression with the Patient Health Questionnaire 9 (PHQ-9) (Kroenke et al., 2001), and symptoms suggestive of anxiety with the Generalised Anxiety Disorder 7 (GAD-7) (Löwe et al., 2008; Spitzer et al., 2006), each referring to the last two weeks. Items were coded 0 (*not at all*), 1 (*several days*), 2 (*more than half the days*), or 3 (*nearly every day*). PHQ-9 scores of 10-27 were taken as suggesting moderate or severe depression(Löwe et al., 2004), and GAD-7 scores of 10-21 moderate or severe anxiety. We used binary variables to describe these outcomes in the analysis. Suicidal thinking was assessed with the question, “In the past 12 months, did you ever consider attempting suicide?”(McKinnon et al., 2016).

The selection of questions to describe emotional, physical, and sexual abuse by an intimate partner or other family member is described elsewhere (Daruwalla et al., 2020). Five questions described emotional violence (insulted or made to feel bad about herself; ignored or treated indifferently; belittled or humiliated in front of others; scared or intimidated on purpose; threats to hurt her or someone close or take her child away), physical violence by nine (pushed, shoved, shaken, hurt; twisted arm, banged head, pulled hair; slapped, pinched, bitten; hit, punched; kicked, dragged, beaten; things thrown at, burned; attacked or threatened with sharp objects or blunt objects; suffocated, choked, hung, poisoned), and sexual violence by four (forced intercourse; forced other degrading act; threatened other act; forced to replicate pornography). Women’s affirmative response to any of these questions—lifetime or past year—was described by binary composite variables for physical violence, sexual violence, and emotional violence.

Cronbach’s alpha indicated internal consistency for the PHQ-9 (α 0.86), GAD-7 (α 0.84), nine items on physical abuse (α 0.83), four items on sexual abuse (α 0.76), five items on emotional abuse (α 0.82), and 24 items on coercive control (α 0.80).

### Statistical Analysis

We tabulated frequencies and proportions of demographic and socioeconomic variables and responses to questions about coercive control, the experience of physical, sexual, and emotional violence, depression, anxiety, and suicidal thinking. Associations between coercive control and other forms of violence were examined by cross-tabulation, followed by univariable and multivariable logistic regression models. We examined the association of coercive control (determinant) with moderate or severe depression, moderate or severe anxiety, and suicidal thinking in the last 12 months (outcomes) in a series of univariable and multivariable logistic regression models. We computed unadjusted and two adjusted models: the first multivariable logistic regression model (aOR1) was adjusted for respondent age, education, religion, caste, asset quintile, respondent and husband employment, respondent and husband drug or alcohol use. The second model (aOR2) was adjusted for other forms of domestic violence and the first model variables (aOR1).

We did two additional analyses. First, we examined the effect of increasing numbers of positive responses to coercive control questions on moderate or severe depression, moderate or severe anxiety, and suicidal thinking in the last 12 months. We adjusted the logistic regression models in the same way as above and then predicted marginal effects and modelled the log-odds of common mental disorder as a step function from 0 to 1 act of control, followed by a linear increase from 1 to 24 acts. We tested for non-linearity by fitting a quadratic term for the increase from 1 to 24.

Second, we analysed possible coercive control sources: either intimate partner or other marital family members. Of the 24 items in our questionnaire, 16 that made this distinction were available. We replicated the analyses described above using this smaller number of coercive control indicators, recategorising the exposure and allowing for effect modification by an intimate partner, a marital family member, or both. All estimates accounted for survey design, with the cluster as the primary sampling unit, four larger areas as strata, and standard errors estimated by Taylor linearisation using *svy* commands in STATA 15.0 (StataCorp LLC).

### Ethical Considerations

Ethical approval was granted by the UCL Research Ethics Committee (3546/003, 27/09/2017) and by PUKAR (Partners for Urban Knowledge, Action, and Research) Institutional Ethics Committee (25/12/2017). The trial before which the data were collected is registered with the Controlled Trials Registry of India (CTRI/2018/02/012047) and ISRCTN (ISRCTN84502355).

## Results

Table 1 summarises the characteristics of 4906 ever-married women aged 18-49 years. Around 19% had no schooling and 38% had reached middle school. A quarter of women were in remunerated work—although 20% of them earned less than INR 12,000 a year (USD 163)—and 98% of their partners were in remunerated work with a mean annual income of INR 172,383 (USD 2335). More than half identified as of general caste. 12% said that they used alcohol or drugs, compared with 44% of their husbands.

Table 2 summarises the prevalence of coercive controlling behaviour, domestic violence, and selected common mental disorders. Overall, 71% of women reported experiencing at least one of the 24 items. The most commonwas that their socialisation, mobility, and access to resources were restricted. Widowed, divorced, or separated women had greater odds of experiencing coercive control than currently married women (adjusted odds ratio 3.2; 95% CI 1.6, 6.8). Forms of violence other than coercive control were also common: 23% reported domestic violence in the last year, of which emotional violence was the most common. Overall, 9% of women screened positive for moderate or severe depressive symptoms on the PHQ-9, 6% for anxiety on the GAD-7, and 6% reported suicidal thinking in the last year.

Crude and both adjusted logistic regression models suggested that women who reported coercive control had greater odds of experiencing emotional (adjusted odds ratio: 2.1; 95% CI 1.7, 2.7), physical (1.4; 1.0, 1.9), and sexual (1.8; 1.1, 3.0) violence in the last 12 months. Table 3 shows associations of coercive control and emotional, physical, and sexual domestic violence with positive screens for depression, anxiety, and reported suicidal thinking. Adjusted models suggested that reported coercive control was associated with greater odds of a positive screen for moderate or severe depression (aOR2 1.7; 1.3, 2.2), independently of the three other forms of domestic violence. Similar findings were seen associating reported coercive control with a positive screen for moderate or severe anxiety (aOR2 2.1; 1.3, 3.1) and suicidal thinking (aOR2 1.7; 1.2, 2.3). Emotional violence independently increased the odds of a positive screen for depression, anxiety, or suicidal thinking three-to-four-fold.

**Figure 1:**
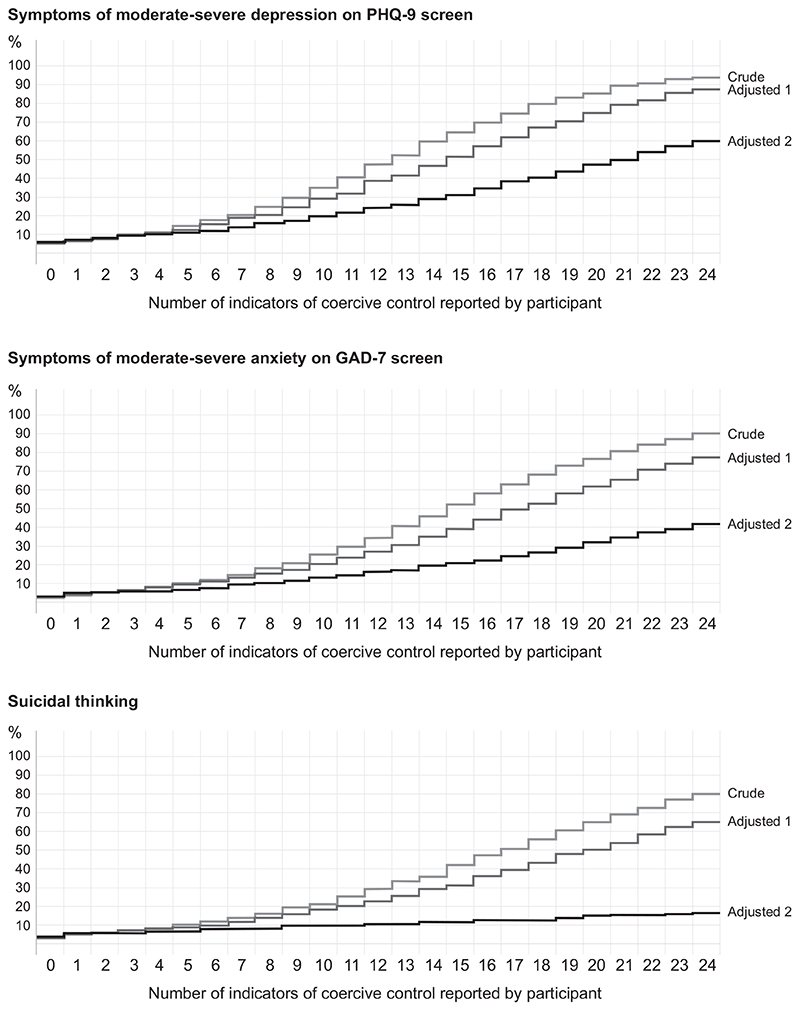
The proportion of women with moderate-severe depression on the PHQ-9 screen, moderate-severe anxiety on the GAD-7 screen, or suicidal thoughts or action, conditional on the experience of 0-24 forms of coercive control behaviour.

Figure 1 shows the effects of coercive control on depression, anxiety, and suicidal thinking based on conditional logistic regression models. For each outcome, predicted marginal effects are presented for three models: crude, adjusted with sociodemographic covariates, and adjusted with both sociodemographic covariates and covariates describing the other three forms of violence. The fully adjusted model showed that, in the absence of coercive behaviour, the predicted proportion of women with depression was 6%, with anxiety 3%, and with suicidal thinking 4%. These proportions increased for each additional indicator of coercive control that women reported. When women reported 24 indicators of coercive controlling behaviour, the predicted proportion with depression was 94% (60% in the second adjusted model), with anxiety 90% (42%), and with suicidal thinking 80% (17%).

We repeated the analysis to distinguish between coercive control by an intimate partner or another marital family member. In this case, the variable describing coercive control was based on 16 questions rather than 24. The odds of a positive screen for moderate or severe depression or anxiety were higher when coercive control was exercised by an intimate partner (aOR2 2.8; 95% CI 2.0, 4.0 for depression, 2.5; 1.5, 4.0 for anxiety) than by a marital family member (1.8; 1.3, 2.5 for depression, 1.7; 1.1, 2.7 for anxiety). They were greatest when respondents reported that coercive control came from an intimate partner and marital family: aOR2 2.8 (95% CI 2.0, 3.9) for depression and 2.8 (1.7, 4.6) for anxiety.

## Discussion

In a survey of over 4000 ever-married women aged 18-49 years in informal settlements in Mumbai, 71% reported at least one form of domestic coercive control. Coercive control was independently associated with double the odds of positive screens for moderate or severe depression, moderate or severe anxiety, and suicidal thinking. The odds of these increased with each additional form of coercive control a woman reported. Coercive control by an intimate partner appeared to have a more substantial influence on depression and anxiety than control by a marital family member. The focus group discussions with counsellors, lawyers and community actors corroborated the results of the study. The themes of non-recognition of coercive control behaviours strains on mental health, and more propensity to abuse and violence emerged from the focus group discussions. The insidious and subtle nature of coercive control tactics made it harder for domestic violence survivors to recognize and deal with it.

Coercive control is a critical element of domestic violence. It makes possible, legitimises, and reinforces other forms of violence by limiting women’s access to resources, harming their self-esteem, self-efficacy, and mental health, and isolating them or reducing their social support (Sowislo & Orth, 2013; Stark, 2007; Thompson et al., 2002). Our results align with the feminist view that spouses commonly use controlling behaviour to subjugate women (Ali & Naylor, 2013; McPhail et al., 2007; Yllo, 2005). At 71%, the prevalence of coercive control in our study was high. Two possible reasons for this—apart from its ubiquity—are that we captured control by intimate partners and marital family members and that asking questions about specific behaviours might be more likely to elicit positive responses than asking more generally about something that might not be thought of as abusive in a situation in which it accords with gender norms.

The association of coercive control with emotional, physical, and sexual violence in the past year was consistent with other studies (Aizpurua et al., 2021; Biswas et al., 2017; Dalal & Lindqvist, 2010; Donta et al., 2015; Gage & Hutchinson, 2006; Krantz & Vung, 2009; Mandal & Hindin, 2013; Mukherjee & Joshi, 2019; Ram et al., 2019). Links between intimate partner violence and various socially controlling behaviours have been found cross-culturally (Russo & Pirlott, 2006), and the risk of violence has been described as increasing with the number of controlling behaviours across diverse cultures (Kishor & Johnson, 2004). Representative studies from Thailand, Nepal, Nigeria, and Turkey have found that marital control by husbands increased the likelihood of spousal violence (Antai, 2011; Chuemchit et al., 2018; Gautam & Jeong, 2019; Yüksel-Kaptanoğlu et al., 2012). Studies in England and the USA found that emotional abuse and marital controlling behaviour were risk factors for physical and sexual intimate partner violence (Felson & Messner, 2000; Hamby & Sugarman, 1999). A second important finding was that coercive control was associated independently with mental health concerns (Abbott et al., 1995; Bergman & Brismar, 1991; Frye et al., 2006; Leone, 2010; Wolford-Clevenger & Smith, 2017; Wolford-Clevenger et al., 2016). It effectively doubled the odds of depression, anxiety, and suicidal thinking, the odds increasing with each additional item in a way generally consistent with previous studies in India (Ahuja et al., 2000; Indu et al., 2017; Nur, 2012; Stephenson et al., 2013; Vachher & Sharma, 2010; Varma et al., 2007). Coercive control targets the survivor’s autonomy, equality, liberty, social support, and dignity in ways that compromise her capacity for independent, self-interested decision-making vital to escape or resist (Stark, 2012). Constraining a woman’s social networks and using psychologically abusive tactics harms her physical and psychological well-being and wears down her will and ability to resist. Separation from family and friends may create a sense of futility and despair. When resistance is lower, compliance with coercive demands may be more likely since there are fewer resources to combat the pressure to comply (Dutton et al., 2006; Stark, 2012). This constrained daily life experience increases the risk of severe injury (Stark, 2007) and contributes to harms to mental health that may be more than those caused by physical violence (Daruwalla et al., 2020; Wolford-Clevenger et al., 2017).

Our focus on coercive control does not imply that the other forms of violence are unimportant: it is part of a matrix of abuse (Myhill & Hohl, 2016). Offenders can subjugate and entrap victims without the use of physical violence, recognising that their controlling tactics will not be taken seriously (Hester & Westmarland, 2006). Controlling tactics, however, predict a range of harms, including sexual, physical and fatal violence, better than prior assault (Beck & Raghavan, 2010; Glass et al., 2004). Adopting the coercive control model would broaden our understanding of partner abuse to resemble most survivors’ experience and improve intervention. Advocacy might encourage a legal view of coercive control as a “liberty crime” (Johnson, 2006; Stark, 2007).

## Limitations

In the absence of a valid measure of coercive control, we asked 24 questions designed to describe a spectrum of coercive controlling behaviours. For this reason our findings are not directly comparable with those of other studies, althoughsimilar forms of restriction, isolation, and control are recognised as abusive by women in many countries (Butterworth & Westmarland, 2015; Hester et al., 2017), and we used many similar questions (Beck & Raghavan, 2010; Dalal & Lindqvist, 2010; Dutton et al., 2006; Mukherjee & Joshi, 2019; Stark & Hester, 2019; Williamson, 2010; Wolford-Clevenger et al., 2017). Our estimates of prevalence may have been increased by the emphasis on sampling younger women and achieving representation of women with disability and the cross-sectional study design means that we are unable to make causal inferences. Of particular note is the (probably) bidirectional relationship between forms of domestic violence and mental health. We cannot say whether domestic violence was the cause or effect of disturbed mental health. Nor did our models include information on the mental health of intimate partners or other family members. Informal settlements may themselves influence the risk of domestic violence and poor mental health in ways for which we were unable to adjust.

## Diversity

The diversity of our study was limited as it involved women aged 18-49 years living in informal settlements. For this reason, the sample represented poorer women and did not involve children, men, or women over the age of 50Transgender women were included and we encouraged inclusion of people with disability.

## Conclusion

Our study considered coercive control as part of the spectrum of domestic violence. It contributes to the disproportionately small evidence base from low- or middle-income settings and considers violence by intimate partners and other family members. Coercive control appears to be a major component of domestic violence and an independent risk factor for depression, anxiety, and suicidal thinking. Understandably, the current focus is on physical and sexual violence, but this needs to expand to consider the controlling behaviours that are often apparent before the physical injury.

For policy and practice, there is enough evidence that the harms of coercive control are as devastating as physical and sexual violence; what is lacking is recognition in legal and healthcare systems. Coercive control needs to be recognised by both policymakers and practitioners as a central feature of domestic violence. It does not feature in India’s Protection of Women from Domestic Violence Act, 2005 (Government of India, 2005). Although the Act was designed to protect women from future domestic violence, it only acknowledges the importance of economic abuse and dowry violence and does not recognize fully that emotional abuse and coercive control limit women’s access to social support, services, and resources. Practitioners need to be aware that women experiencing coercive control require social, legal, and medical attention. Early intervention will protect some women from progression to, for example, physical domestic violence, as well as from harms to mental health and isolation.

## Figures and Tables

**Table 1:** Characteristics of 4906 Ever-married Women Respondents in Informal Settlements in Mumbai, India

**Marital status**	(n)	(%)
Currently married	4694	(96)
Widowed/Separated/Divorced	212	(4)
**Respondent age (in complete years)**		
18-25 y	1025	(21)
26-30 y	1421	(29)
31-36 y	1172	(24)
37-49 y	1288	(26)
**Respondent education**		
No education	938	(19)
Primary 1-5 y	846	(17)
Middle 6-8 y	1099	(22)
High 9-10 y	1105	(23)
Senior 11-12 y	533	(11)
Above 12 y	385	(8)
**Respondent employed**	1182	(24)
**Respondent monthly income, INR**		
<1000	233	(20)
1000-2999	303	(27)
3000-5999	279	(25)
6000+	322	(28)
**Respondent uses alcohol or drugs**	612	(12)
**Husband age**		
18-19 y	14	(<1)
20-29 y	917	(19)
30-39 y	2102	(44)
40-49 y	1370	(29)
50+ y	391	(8)
**Husband employed**	4686	(98)
**Husband monthly income, INR**		
<10,000	1095	(23)
10,000-11,999	997	(21)
12,000-14,999	652	(14)
15,000+	1942	(41)
**Husband uses alcohol or drugs**	2100	(44)
**Housing type**		
Kachha (insubstantial)	336	(7)
Pukka (robust)	2518	(51)
Mixed	2052	(42)
**Toilet type**		
Private	836	(17)
Public	4368	(82)
Open defecation	2	(<1)
**Religion**		
Hindu	1826	(37)
Muslim	2882	(59)
Other	198	(4)
**Caste**		
General	2854	(58)
OtherBackward Caste	1180	(24)
Scheduled Tribe or Caste	872	(18)
**Socioeconomic quintile**		
1 poorest	969	(21)
2	936	(20)
3	934	(20)
4	933	(20)
5 least poor	935	(20)
**All**	**4906**	**(100)**

**Table 2:** Prevalence of Coercive Controlling Behaviour, Domestic Violence, and Common Mental Disorders among 4906 Ever-married Women Respondents in Informal Settlements in Mumbai, India

	**(n)**	**(%)**
**Coercive control behaviour**	**3465**	**(71)**
Dress or hairstyle dictated by others	256	(5)
Excluded from family matters	357	(7)
Needs permission for healthcare	711	(14)
Limited access to household areas	168	(3)
Forced out of house	162	(3)
Locked in house	37	(1)
Prevented from attending meetings	220	(4)
Movement monitored	386	(8)
Prevented from seeking employment	825	(17)
Coerced to seek employment	76	(2)
Prevented from schooling	148	(3)
Given excessive work	236	(5)
Coerced to use contraception	15	(<1)
Prevented from using contraception	52	(1)
Prevented from terminating pregnancy	37	(1)
Coerced to terminate pregnancy	14	(<1)
Never free to talk on phone	638	(13)
Never free to speak	501	(10)
Needs permission to go out	1245	(25)
Accompanied when out	510	(10)
Never allowed out in evening	652	(13)
Can never meet female friends	488	(10)
Can never meet male friends/acquaintances	2388	(49)
Can never meet natal family	249	(5)
**Domestic violence in last 12 months**	**1104**	**(23)**
Physical violence	618	(13)
Sexual violence	186	(4)
Emotional violence	927	(19)
**Domestic violence (lifetime)**	**1877**	**(38)**
Physical violence	1243	(25)
Sexual violence	285	(6)
Emotional violence	1553	(32)
**Common mental disorder**		
Moderate or severe depression on PHQ-9	443	(9)
Moderate or severe anxiety on GAD-7	299	(6)
Suicidal thinking in last 12 months	318	(6)
**All**	**4906**	**(100)**

PHQ-9: Patient Health Questionnaire 9-question screen. GAD-7: Generalised Anxiety Disorder 7-question screen.

**Table 3:** Association of Coercive Control Behaviour with Depression, Anxiety, and Suicidal thinking among 4906 Ever-married Women Respondents in Informal Settlements in Mumbai, India

		No	(%)	Yes	(%)	OR [95% CI]	OR_1_ [95% CI]	OR_2_ [95% CI]
**Outcome: Moderate or severe depression on PHQ-9 screen**								
***Exposures***								
Coercive control behaviour								
	No	1368	(95)	73	(5)	1	1	1
	Yes	3095	(89)	370	(11)	2.2 [1.8, 2.8]	2.2 [1.8, 2.9]	1.7 [1.3, 2.2]
Emotional violence in Last 12 m								
	No	3759	(94)	220	(6)	1	1	1
	Yes	704	(76)	223	(24)	5.4 [4.4, 6.7]	4.8 [3.9, 6.0]	3.3 [2.5, 4.3]
Physical violence in last 12 m								
	No	3996	(93)	292	(7)	1	1	1
	Yes	467	(76)	151	(24)	4.4 [3.5, 5.6]	4.0 [3.1, 5.2]	1.5 [1.1,2.1]
Sexual violence in last 12 m								
	No	4336	(92)	384	(8)	1	1	1
	Yes	127	(68)	59	(32)	5.3 [3.8, 7.3]	4.5 [3.1,6.5]	1.8 [1.3, 2.7]
**Outcome: Moderate or severe anxiety on GAD-7 screen**								
***Exposures***								
Coercive control behaviour								
	No	1400	(97)	41	(3)	1	1	1
	Yes	3207	(93)	258	(7)	2.8 [1.9, 4.0]	2.7 [1.8, 4.1]	2.1 [1.3, 3.1]
Emotional violence in last 12 m								
	No	3843	(97)	136	(3)	1	1	1
	Yes	764	(82)	163	(13)	6.0 [4.6, 7.9]	5.5 [4.1, 7.4]	3.6 [2.6, 5.0]
Physical violence in last 12 m								
	No	4099	(96)	189	(4)	1	1	1
	Yes	508	(82)	110	(18)	4.7 [3.6, 6.2]	4.5 [3.3, 6.1]	1.5 [1.1,2.2]
Sexual violence in last 12 m								
	No	4465	(95)	255	(5)	1	1	1
	Yes	142	(76)	44	(24)	5.4 [3.7, 8.0]	4.9 [3.1, 7.8]	1.9 [1.2, 3.1]
**Outcome: Suicidal thinking in last 12 m**								
***Exposures***								
Coercive control behaviour								
	No	1395	(97)	46	(3)	1	1	1
	Yes	3193	(92)	272	(8)	2.6 [1.9, 3.6]	2.5 [1.8, 3.4]	1.7 [1.2, 2.3]
Emotional violence in last 12 m								
	No	3849	(97)	130	(3)	1	1	1
	Yes	739	(80)	188	(20)	7.5 [5.9, 9.6]	6.8 [5.3, 8.8]	3.4 [2.3, 5.1]
Physical violence in last 12 m								
	No	4119	(96)	169	(4)	1	1	1
	Yes	469	(76)	149	(24)	7.7 [5.9, 10.1]	6.8 [5.0, 9.1]	2.5 [1.6, 3.8]
Sexual violence in last 12 m								
	No	4461	(95)	259	(5)	1	1	1
	Yes	127	(68)	59	(32)	8.0 [5.6, 11.4]	6.5 [4.4, 9.6]	2.2 [1.4, 3.3]

PHQ-9: Patient Health Questionnaire 9-question screen. GAD-7: Generalised Anxiety Disorder 7-question screen. OR: crude odds ratio. aOR1: odds ratio adjusted with covariates for respondent age, education, religion, caste, socioeconomic quintile, respondent and husband employment, respondent and husband drug or alcohol use. aOR2: odds ratio adjusted as aOR1 plus covariates for emotional, physical, and sexual violence.

## Data Availability

Data are available in the Open Science Framework: Suman Kanougiya (2021, February 26). Coercive control dataset. Retrieved from https://osf.io/4y6vd/
